# Deep learning–assisted diagnosis of acute mesenteric ischemia based on CT angiography images

**DOI:** 10.3389/fmed.2025.1510357

**Published:** 2025-01-24

**Authors:** Lei Song, Xuesong Zhang, Jian Zhang, Jie Wu, Jinkai Wang, Feng Wang

**Affiliations:** ^1^Department of Interventional Therapy, The First Affiliated Hospital of Dalian Medical University, Dalian, Liaoning, China; ^2^Department of Interventional Therapy, The Second Affiliated Hospital of Dalian Medical University, Dalian, China

**Keywords:** acute mesenteric ischemia, multiphase CT angiography, artificial intelligence, deep learning, disease diagnosis

## Abstract

**Purpose:**

Acute Mesenteric Ischemia (AMI) is a critical condition marked by restricted blood flow to the intestine, which can lead to tissue necrosis and fatal outcomes. We aimed to develop a deep learning (DL) model based on CT angiography (CTA) imaging and clinical data to diagnose AMI.

**Methods:**

A retrospective study was conducted on 228 patients suspected of AMI, divided into training and test sets. Clinical data (medical history and laboratory indicators) was included in a multivariate logistic regression analysis to identify the independent factors associated with AMI and establish a clinical factors model. The arterial and venous CTA images were utilized to construct DL model. A Fusion Model was constructed by integrating clinical factors into the DL model. The performance of the models was assessed using receiver operating characteristic (ROC) curves and decision curve analysis (DCA).

**Results:**

Albumin and International Normalized Ratio (INR) were associated with AMI by univariate and multivariate logistic regression (*P* < 0.05). In the test set, the area under ROC curve (AUC) of the clinical factor model was 0.60 (sensitivity 0.47, specificity 0.86). The AUC of the DL model based on CTA images reached 0.90, which was significantly higher than the AUC values of the clinical factor model, as confirmed by the DeLong test (*P* < 0.05). The Fusion Model also showed exceptional performance in terms of AUC, accuracy, sensitivity, specificity, and precision, with values of 0.96, 0.94, 0.94, 0.95, and 0.98, respectively. DCA indicated that the Fusion Model provided a greater net benefit than those of models based solely on imaging and clinical information across the majority of the reasonable threshold probabilities.

**Conclusion:**

The incorporation of CTA images and clinical information into the model markedly enhances the diagnostic accuracy and efficiency of AMI. This approach provides a reliable tool for the early diagnosis of AMI and the subsequent implementation of appropriate clinical intervention.

## 1 Introduction

Acute mesenteric ischemia (AMI) is a life-threatening condition characterized by restricted blood flow to the intestine, leading to tissue necrosis and potentially fatal outcomes if not promptly diagnosed and treated (1, 2). The pathophysiological processes underlying AMI are complex, involving embolic, thrombotic, and low-perfusion states that complicate early detection and effective intervention (3, 4). Despite advances in medical imaging and surgical techniques, the morbidity and mortality rates associated with AMI remain alarmingly high (5, 6).

Abdominal pain is the sole presenting symptom of most AMI patients when they seek medical treatment. Some AMI patients present with “severe symptoms and mild signs” in the early stage of the disease, but this clinical picture lacks specificity (7). Therefore, many scholars focus on laboratory test indicators to diagnose whether patients with acute abdomen have AMI, such as white blood cell counts, C-reactive protein, and lactate levels (8–11). However, elevated white blood cells and C-reactive protein levels are typically responses to various infections, inflammation, or stress and are not specific to AMI. While AMI can lead to increased lactate levels, changes in lactate levels may be delayed and might not immediately reflect the condition in the disease’s early stages. Therefore, relying solely on laboratory indicators challenges capturing the complexity of AMI due to their inadequate sensitivity and specificity (12, 13). As the understanding of AMI deepens, developing a comprehensive diagnostic approach that combines laboratory tests and imaging techniques will be crucial for improving diagnostic accuracy and timeliness.

Computed tomography angiography (CTA) stands as a cornerstone in the diagnostic workup of suspected AMI (14). CTA technology enables the clear observation of the extent of mesenteric vascular occlusion and stenosis, as well as the evaluation of their relationship with adjacent branch vessels. Furthermore, CTA can facilitate the assessment of intestinal lesions and mesenteric changes associated with AMI (15). However, the interpretation of CT images is often challenging due to the subtle and variable imaging manifestations of AMI, necessitating significant expertise and may result in delayed or missed diagnoses (16).

Recent advancements in artificial intelligence, particularly deep learning (DL), have demonstrated substantial promise in enhancing diagnostic accuracy and efficiency across a range of medical imaging tasks (17, 18). Moreover, they have exhibited significant advantages in identifying other acute abdominal conditions, such as acute pancreatitis and appendicitis (19, 20).

We hypothesize that DL is able to be utilized in CTA images to build an early diagnostic model for AMI. Additionally, integrating clinical information with DL may provide supplementary data for the diagnostic model. Therefore, it is necessary to develop a more systematic and comprehensive model to achieve multi-level clinical decision-making. This study aims to construct an integrated model utilizing DL and clinical information to provide a reliable tool for early diagnosis in patients with AMI.

## 2 Materials and methods

### 2.1 Subjects

This retrospective study was approved by our ethic institutional review board.

The electronic medical records of 385 adult patients suspected of AMI from 2011 to 2023 were retrieved using a lexicon search tool. This tool identified specific keyword phrases such as “acute mesenteric ischemia,” “bowel ischemia/necrosis,” and “pneumoperitoneum.” The exclusion criteria were as follows: (1) no exploratory laparotomy, no pathological analysis of any resected specimen, or death before receiving the intervention or surgical treatment (79 patients); (2) no multi-phase contrast-enhanced CT scans (45 patients); and (3) missing clinical information or laboratory indicators (33 patients).

A total of 228 patients (about 182,000 CTA images in total, an average of 800 CTA images per patient) were enrolled in this study. All patients were divided into AMI and non-AMI according to verified clinical diagnoses, and randomly assigned to a training set and a test set at a ratio of 7:3. The training set comprised 160 patients, of which 111 were identified as having acute mesenteric ischemia (AMI) and 49 as non-AMI. The test set included 68 patients, with 47 identified as AMI and 21 as non-AMI.

### 2.2 CT examinations

CT examinations were performed on two CT scanners from GE (256 and 64-slice CT). All patients underwent abdominal arterial and venous CTA examinations. Firstly, arterial angiography was performed using bolus tracking technology, with the area of interest located in the descending aorta (threshold = 180 HU). Subsequently, a venous CTA examination was conducted after a 30-s delay. The scan range of all phases was from the diaphragm to the symphysis pubis in a supine position. All patients underwent CT scans at 120 kVp tube voltage and automatic tube current. CT images were reconstructed with a section thickness of 1.25 mm.

### 2.3 Clinical factor model

The comprehensive clinical data for all patients was collected, including the history of hypertension, diabetes, atrial fibrillation, coronary heart disease, previous strokes, liver cirrhosis, lower limb venous thrombosis, average pulse rate, and systolic and diastolic blood pressure, along with laboratory indicators such as white blood cell count, hemoglobin, platelet count, lymphocytes, neutrophils, serum creatinine, albumin, fasting blood glucose, alanine aminotransferase (ALT), aspartate aminotransferase (AST), D-dimer, fibrinogen, and International Normalized Ratio (INR). Independent risk factors related to AMI were identified through univariate and multivariate logistic analyses to establish a clinical factor model.

### 2.4 Deep learning model

An automatic segmentation algorithm was employed to delineate 3D volumes of interests (VOIs) for the superior mesenteric artery (SMA), small intestine, colon, and abdominal fat were delineated on arterial phase images, as well as VOIs for the portal vein, superior mesenteric vein, small intestine, colon, and abdominal fat on venous phase images. In total, eight VOIs were outlined for each patient. To ensure accuracy, two radiologists with 10 years of experience in abdominal imaging reviewed all VOIs while being blinded to clinical and pathological information. The labels for the images were based on verified clinical diagnoses, categorizing them into acute mesenteric ischemia (AMI) and non-AMI groups. These labels served as the ground truth for model training and evaluation.

In the data preprocessing stage, all images were resampled with voxel spacing of 1 × 1 × 1 mm^3^ and normalized by Z-score normalization. After cropping the borders of the 8 VOIs to 32 × 32 × 32, they are input into the network for subsequent analysis. To improve data diversity and the generalization ability of the model, data augmentation techniques were employed. These included horizontal flip, vertical flip, and transposition.

In the training data set, we used 3D convolutional neural network ResNet50 to establish 9 deep learning models for diagnosing AMI and non-AMI. These models are as follows, *Model A*: based on the VOI of SMA in arterial phase; *Model B*: based on the VOI of small intestine in arterial phase; *Model C*: based on the VOI of colon in arterial phase; *Model D*: based on the VOI of abdominal fat in arterial phase; *Model E*: based on the VOI of vein in venous phase; *Model F*: based on the VOI of small intestine in venous phase; *Model G*: based on the VOI of colon in venous phase; *Model H*: based on the VOI of abdominal fat in venous phase; and *Model I*: a comprehensive model integrating all VOIs.

The ResNet50 architecture contained 16 residual blocks. Each residual block consisted of three convolutional layers (1 × 1, 3 × 3, and 1 × 1), batch normalization layer and rectified linear unit activation function. The output of the last block was connected to a fully-connected layer and a softmax layer to give the probability for diagnosis of AMI and non-AMI. To enhance the transparency of the models’ decision-making process, Gradient-weighted Class Activation Mapping (Grad-CAM) was employed to visualize the models. By utilizing gradient information from the last convolutional layer for weighted fusion, class activation maps were generated to underscore key regions of the image pertinent to classification targets (21).

Configuration: The batch size was set to 8, and the IO threads were set to 4. The Adam training optimizer was used with a learning rate set to 0.0001. The hardware was a GeForce RTX 2080 Ti GPU, and the software included Python 3.7.11 and PyTorch 1.7.1.

### 2.5 Fusion Model

**FIGURE 1:**
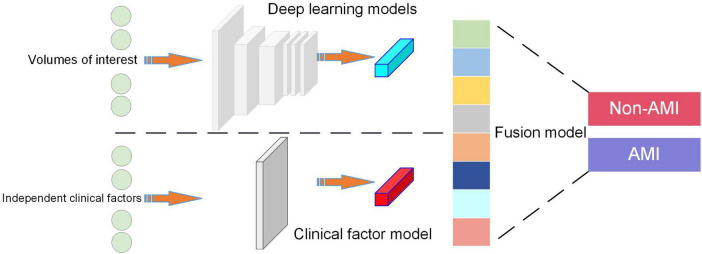
Schematic diagram of the Fusion Model construction.

To obtain DL fused clinical factor model for diagnosing the probabilities of AMI and non-AMI, the independent clinical risk factors were integrated into the fully-connected layer of *Model I*. Construction process seen in Figure 1.

### 2.6 Statistical analysis

Statistical analyses were performed using SPSS version 24.0 and Python version 3.7.11. The normality of the measurement data was assessed with the Kolmogorov-Smirnov test (*n* > 50) or Shapiro-Wilk test (*n* ≤ 50). Data that followed a normal distribution were expressed as mean ± standard deviation and compared between AMI and non-AMI patients using two-sample *t*-tests. Data that not followed a normal distribution were expressed as median (Q1, Q3) and compared using the Mann-Whitney U test. Count data were presented as frequencies and percentages, and comparisons were made using the Chi-square test.

Independent risk factors related to AMI were identified through univariate and multivariate logistic regression analyses to establish a clinical factor model. First, the univariate logistic regression analysis was conducted to screen for potential risk factors by calculating odds ratios (ORs) with a corresponding 95% confidence interval (CI) for each variable. Variables with a *p*-value of less than 0.05 in the univariate analysis were considered eligible for inclusion in the multivariate logistic regression model. For the multivariate logistic regression analysis, the backward stepwise selection method was employed to further refine the model. This method begins with the inclusion of all candidate variables identified from the univariate analysis and subsequently removes variables that do not contribute significantly to the model.

The diagnostic efficiency was quantified using the area under the receiver operating characteristic (ROC) curve and then the area under the ROC curves (AUCs) were calculated. The AUCs were compared using the DeLong test. Specific performance metrics, including sensitivity, specificity, accuracy, precision, and the F1 score, were calculated. To assess clinical usefulness of the models, a decision curve analysis (DCA) was conducted to evaluate the clinical net benefit. Additionally, a calibration curve was plotted to assess the model’s reliability. A *p*-value less than 0.05 was considered statistically significant.

## 3 Results

### 3.1 Clinical data

Table 1 lists the details of the clinical data of all patients. There were no significant differences between the AMI group and the non-AMI group.

**TABLE 1:** The details of the baseline characteristics and clinical data of all individuals.

Characteristics	Training set (*n* = 160)	Test set (*n* = 68)	*P*-value
Age (years)			0.725^†^
Median (IQR)	71 (64, 79)	68 (65, 75)	
Gender (no.)			0.905*
Female	9	6	
Male	45	28	
AMI (no.)			0.969*
Yes	111	47	
No	49	21	
Hypertension (no.)			0.398*
Yes	78	29	
No	82	39	
Diabetes (no.)			0.582*
Yes	28	14	
No	132	54	
Atrial fibrillation (no.)			0.136*
Yes	43	12	
No	117	56	
Coronary heart disease (no.)			0.490*
Yes	27	9	
No	133	59	
Previous strokes (no.)			0.063*
Yes	18	14	
No	142	54	
Liver cirrhosis (no.)			0.941*
Yes	5	2	
No	155	66	
Lower limb venous thrombosis (no.)			0.814*
Yes	6	3	
No	154	65	
Average pulse rate (bpm)	78 (70, 90)	78 (74, 92)	0.15^†^
Systolic blood pressure (mmHg)	138 (122, 150)	136 (124, 149.25)	0.548^†^
Diastolic blood pressure (mmHg)	80 (76, 90)	85 (79.25, 90.75)	0.31^†^
White blood cell count (cells/μL)	8.81 (5.79, 13.58)	9.385 (6.11, 14.1575)	0.458^†^
Platelet count (platelets/μL)	131 (118.25, 144.75)	134.5(123, 145.25)	0.52^†^
Hemoglobin (g/dL)	192.5 (150.3, 235)	198.5 (157.75, 246.75)	0.458^†^
Neutrophils (cells/μL)	1.445 (0.87, 2.05)	1.415 (0.9025, 1.83)	0.598^†^
Lymphocytes (cells/μL)	6.76 (3.98, 11.52)	6.74 (3.96, 12.115)	0.749^†^
Albumin (g/dL)	68 (55, 94.25)	72.5 (55.25, 87.75)	0.751^†^
Serum creatinine (mg/dL)	37.4 (32.95, 41.075)	37.2 (34.525, 39.55)	0.585^†^
ALT (U/L)	6.185 (5.24, 9.16)	5.955 (4.9625, 8.3825)	0.136^†^
Fasting blood glucose (mg/dL)	20.5 (15, 30.5)	22.5 (15, 30)	0.279^†^
AST (U/L)	23 (18, 34.75)	25 (19.25, 34)	0.365^†^
D-dimer (ng/mL)	1.6 (0.61, 4.46)	1.895 (0.5925, 4.225)	0.646^†^
Fibrinogen (mg/dL)	3.56 (2.84, 4.61)	3.835 (2.8225, 4.8425)	0.285^†^
INR	1.05 (0.98, 1.16)	1.03 (0.97, 1.16)	0.443^†^

ALT, alanine aminotransferase; INR, International Normalized Ratio; AST, aspartate aminotransferase.

*The Chi-square test.

^†^The Mann-Whitney U test.

### 3.2 Performance of clinical factor model

In the training set, Albumin and INR were associated with AMI by univariate and multivariate logistic regression (*P* < 0.05) (Table 2). In the test set, the AUC of the clinical factor model was 0.60 (sensitivity 0.47, specificity 0.86). Additional detailed indicators were presented in Table 3.

**TABLE 2:** Risk factors for diagnosing AMI and non-AMI in the training set.

Univariate logistic regression					
**Variables**	**β**	**S.E**	***Z***	***P***	**OR (95%CI)**
Hypertension	0.46	0.35	1.33	0.184	1.59 (0.80 ∼ 3.13)
Diabetes	0.12	0.46	0.26	0.795	1.13 (0.46 ∼ 2.77)
Atrial fibrillation	1.06	0.46	2.32	**0.020**	2.88 (1.18 ∼ 7.04)
Coronary heart disease	0.28	0.48	0.58	0.562	1.32 (0.52 ∼ 3.36)
Previous strokes	0.48	0.59	0.81	0.415	1.62 (0.51 ∼ 5.21)
Liver cirrhosis	0.58	1.13	0.52	0.605	1.79 (0.20 ∼ 16.48)
Lower limb venous thrombosis	15.80	979.61	0.02	0.987	7303968.37 (0.00 ∼ Inf)
Average pulse rate	0.01	0.01	1.46	0.143	1.01 (1.00 ∼ 1.03)
Systolic blood pressure	0.02	0.01	2.36	**0.018**	1.02 (1.01 ∼ 1.04)
Diastolic blood pressure	0.00	0.01	0.01	0.991	1.00 (0.97 ∼ 1.03)
White blood cell count	0.07	0.03	2.23	**0.026**	1.08 (1.01 ∼ 1.15)
Platelet count	−0.00	0.00	−1.12	0.263	1.00 (0.99 ∼ 1.00)
Hemoglobin	0.01	0.01	0.87	0.385	1.01 (0.99 ∼ 1.02)
Neutrophils	0.05	0.03	1.84	0.066	1.05 (1.00 ∼ 1.11)
Lymphocytes	−0.10	0.08	−1.32	0.188	0.90 (0.78 ∼ 1.05)
Albumin	−0.11	0.03	−3.40	**<0.001**	0.90 (0.84 ∼ 0.96)
Serum creatinine	0.00	0.00	0.22	0.825	1.00 (1.00 ∼ 1.01)
ALT	0.01	0.01	1.04	0.300	1.01 (0.99 ∼ 1.03)
Fasting blood glucose	0.08	0.05	1.46	0.145	1.08 (0.97 ∼ 1.20)
AST	0.01	0.01	1.34	0.179	1.01 (0.99 ∼ 1.03)
D-dimer	0.08	0.05	1.69	0.091	1.08 (0.99 ∼ 1.18)
Fibrinogen	0.28	0.13	2.15	**0.032**	1.33 (1.03 ∼ 1.72)
INR	5.91	1.73	3.41	**<0.001**	369.83 (12.40 ∼ 11029.74)
**Multivariate logistic regression**					
Variables	β	S.E	*Z*	*P*	OR (95%CI)
Atrial fibrillation	0.36	0.52	0.69	0.493	1.43 (0.52 ∼ 3.94)
Systolic blood pressure	0.02	0.01	1.69	0.092	1.02 (1.00 ∼ 1.04)
White blood cell count	0.03	0.04	0.91	0.361	1.03 (0.96 ∼ 1.11)
Albumin	−0.08	0.04	−2.32	**0.021**	0.92 (0.86 ∼ 0.99)
Fibrinogen	0.14	0.15	0.90	0.370	1.15 (0.85 ∼ 1.56)
INR	4.01	1.67	2.41	**0.016**	55.38 (2.10 ∼ 1458.26)

ALT, alanine aminotransferase; INR, International Normalized Ratio; AST, aspartate aminotransferase; OR, odds ratio; CI, confidence interval, IQR, Interquartile Range. Values with a statistically significant difference (*p*-value < 0.05) are in bold.

**TABLE 3:** The performance of different models.

Set	Model	AUC	Accuracy	Sensitivity	Specificity	F1 Score	Precision
Training set	Model A	0.89	0.83	0.80	0.88	0.86	0.94
	Model B	0.86	0.81	0.83	0.75	0.86	0.88
	Model C	0.79	0.73	0.73	0.71	0.79	0.85
	Model D	0.85	0.73	0.61	0.97	0.76	0.98
	Model E	0.74	0.83	0.95	0.55	0.88	0.83
	Model F	0.80	0.83	0.86	0.73	0.88	0.89
	Model G	0.75	0.71	0.72	0.69	0.78	0.84
	Model H	0.80	0.75	0.75	0.78	0.82	0.88
	Model I	0.94	0.93	0.90	0.97	0.91	0.99
	Clinical model	0.72	0.66	0.62	0.76	0.72	0.85
	Fusion Model	0.99	0.98	0.98	0.98	0.99	0.98
Test set	Model A	0.88	0.85	0.89	0.75	0.89	0.89
	Model B	0.81	0.87	0.96	0.67	0.91	0.87
	Model C	0.76	0.75	0.68	0.90	0.79	0.94
	Model D	0.80	0.82	0.91	0.62	0.88	0.84
	Model E	0.70	0.78	0.38	0.80	0.86	0.78
	Model F	0.77	0.85	0.89	0.76	0.87	0.89
	Model G	0.72	0.71	0.66	0.81	0.76	0.89
	Model H	0.77	0.82	0.85	0.76	0.87	0.89
	Model I	0.90	0.95	0.90	0.97	0.89	0.95
	Clinical model	0.60	0.59	0.47	0.86	0.61	0.88
	Fusion Model	0.96	0.94	0.94	0.95	0.96	0.98

### 3.3 Performance of DL model

Table 3 summarized the performances of different DL models. The AUC values for Model A, Model B, Model D, Model F and Model H in the training set all >0.80. The AUC values were 0.89 (sensitivity 0.80, specificity 0.88), 0.86 (sensitivity 0.83, specificity 0.75), and 0.85 (sensitivity 0.61, specificity 0.97), 0.80 (sensitivity 0.86, specificity 0.83), and 0.80 (sensitivity 0.75, specificity 0.78), respectively.

**FIGURE 2:**
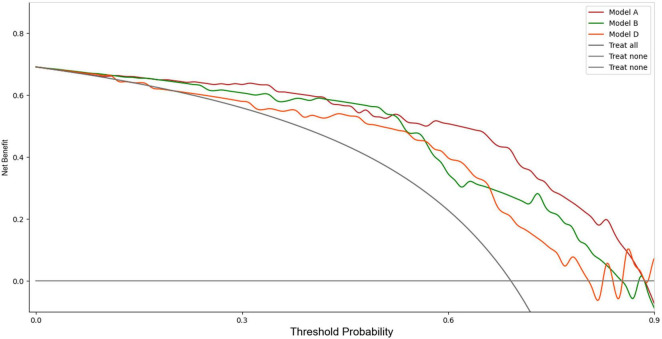
DCA of the Model A, Model B and Model D in the test set.

**FIGURE 3:**
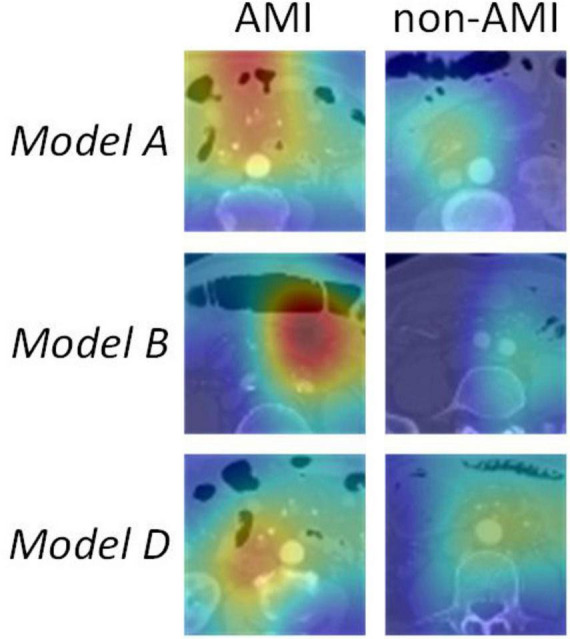
The attention regions of deep learning models in CTA image analysis.

Upon validation of the models on the test set, only Model A, Model B, and Model D had AUCs > 0.8. The AUC values were 0.88 (sensitivity 0.89, specificity 0.75), 0.81 (sensitivity 0.96, specificity 0.67), and 0.80 (sensitivity 0.91, specificity 0.62), respectively. The DCA indicated that within the majority of reasonable threshold probability ranges, Model A had a higher overall net benefit than Model B and Model D. This suggests Model A is more consistent in distinguishing between AMI and non-AMI compared to Model B and Model D (Figure 2). In terms of model interpretability, the attention regions of Model A, Model B, and Model D were all activated, and there were significant differences in distinguishing AMI and non-AMI (Figure 3).

### 3.4 Assessment of the performance of the different models

To enhance diagnostic performance, DL information was integrated to develop Model I. Subsequently, we combined DL with clinical information to create the Fusion Model. Validation results indicated that Model I improved the accuracy of AMI diagnosis, while the Fusion Model further enhanced diagnostic efficiency over Model I.

**FIGURE 4:**
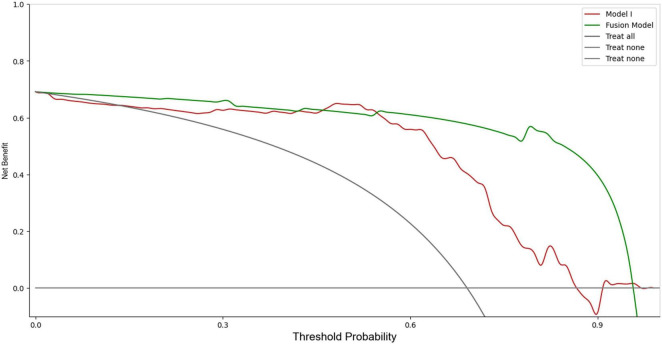
DCA of the Model I and Fusion Model in the test set.

In the training set, Model I achieved an AUC of 0.94 (sensitivity 0.90, specificity 0.97), which was higher than those of models based solely on imaging and clinical information. The Fusion Model exhibited the best performance, with an AUC of 0.99 (sensitivity 0.98, specificity 0.98), outperforming Model I. In the test set, the AUC of Model I slightly decreased but still reached 0.90, which remained higher than the AUC values of the imaging and clinical models, as confirmed by the DeLong test (*P* < 0.05). The Fusion Model also demonstrated exceptional performance in terms of AUC, accuracy, sensitivity, specificity, and precision, with values of 0.96, 0.94, 0.94, 0.95, and 0.98, respectively. Within the threshold probability range of 0.6–0.95, the clinical net benefit of Fusion Model was higher than that of Model I (Figure 4).

## 4 Discussion

In this study, we constructed an early diagnosis DL model for AMI by integrating imaging and clinical information. The results revealed that our Fusion Model significantly outperformed individual models based on clinical or imaging data alone. Specifically, the Fusion Model achieved an impressive AUC of 0.96 in the test set, alongside high values in sensitivity, specificity, accuracy, and precision. These findings indicate that using advanced DL technology to integrate imaging information and clinical data can significantly improve diagnostic performance, enabling earlier and more accurate diagnosis of AMI in clinical settings.

AMI is defined as a sudden occlusion or reduction in blood flow to the intestines, resulting in tissue ischemia and necrosis (22). Early diagnosis is critical for patient survival yet, clinical symptoms are often nonspecific and may overlap with other abdominal emergencies (23). In this study, the clinical factor model alone showed limited diagnostic performance, with an AUC of only 0.60 in the test set. Despite the fact that albumin and INR were significantly associated with AMI in univariate and multivariate regression analyses, relying solely on clinical indicators proved inadequate for achieving satisfactory diagnostic efficacy. This finding highlights the complex pathophysiological characteristics of AMI, which likely requires a multi-faceted approach combining various data types to attain accurate diagnostic flagging (24).

CTA has become the preferred test in diagnosing AMI due to its ability to provide comprehensive vascular and parenchymal information (25). The multi-phase nature of CTA facilitates capturing both arterial and venous phases, which are crucial for the detection of vascular occlusions and the identification of areas of ischemia (26). Despite its utility, the interpretation of CTA images remains a complex and demanding process, often necessitating the expertise of seasoned radiologists (27).

Our study explored various models based on distinct VOIs delineated from the superior mesenteric artery (SMA), small intestine, colon, and abdominal fat in both arterial and venous phases. Our findings revealed notable variations in the diagnostic efficacy across these regions. Models A, B, and D-associated with the SMA, small intestine, and abdominal fat, respectively-exhibited a higher diagnostic performance. Specifically, Model A, which focused on the SMA, achieved an AUC of 0.88 in the test set, emphasizing the vital role of arterial phase imaging in detecting AMI. This finding aligns with previous studies highlighting the diagnostic value of arterial phase images in identifying vascular occlusions and reduced perfusion (28, 29). The performance of Model B underscored the critical importance of evaluating the small intestine for ischemic changes. The sensitivity of 0.96 emphasizes its potential for detecting subtle ischemic changes within the bowel wall, such as thickening or loss of enhancement, which are crucial early indicators of AMI (30, 31). Model D, which focused on abdominal fat in the arterial phase, also presented robust performance with an AUC of 0.80. This model exhibited the capacity to detect exudation and other secondary effects of ischemia in the surrounding fat, thereby underscoring its complementary diagnostic value (32, 33).

In contrast, venous phase models such as Model E, F, and G yielded comparatively lower AUCs, indicating that while venous phase images provide valuable supplementary information, they may be less critical than arterial phase images for initial AMI detection. These findings partially diverge from some earlier works that suggest comprehensive evaluations of both arterial and venous phases for diagnosing vascular pathologies (25, 26). Our results indicate, however, that arterial phase images may hold greater diagnostic yield when considered independently.

The discrepancy between the performance of different DL models emphasizes the importance of comprehensive image analysis across multiple anatomical regions to accurately capture the heterogeneous presentation of AMI. Model I, as an integrated model encompassing multiple VOIs, showed a significant improvement in diagnosing AMI compared to models based solely on imaging data. Specifically, Model I achieved an AUC of 0.90 in the test set, highlighting its superiority over single CTA imaging models. To further enhance diagnostic performance, we combined Model I with clinical information to produce the Fusion Model. The Fusion Model, however, demonstrated superior performance to Model I, achieving an AUC of 0.99 in the training set and 0.96 in the test set. It also demonstrated exceptional performance across other metrics. The DCA indicated that the Fusion Model offered the highest clinical net benefit across a range of reasonable threshold probabilities, further validating its potential for clinical application.

The strength of our study mainly includes the following points. Firstly, the integration of arterial and venous phase VOIs provided a more comprehensive anatomical and functional landscape, significantly enhancing the reliability of image recognition. Secondly, advanced deep learning techniques facilitated effective image processing and feature extraction, making automated CTA-based diagnostics feasible. Lastly, the incorporation of clinical information augmented the model’s diagnostic capacity, enhancing its accuracy and suitability for the early diagnosis of AMI.

Several limitations of our study should be acknowledged. Firstly, the retrospective nature of the study may introduce selection bias, despite our efforts to mitigate this through the application of strict inclusion and exclusion criteria. Secondly, the relatively small sample size, especially in the testing set, may affect the reliability of the results and limit the generalizability of our findings. Thirdly, although the DL models demonstrated high performance metrics, external validation with different datasets is required to assess their robustness across diverse clinical settings and populations. Furthermore, the study focuses solely on CTA imaging data and clinical factors, which may result in the exclusion/overlook of CT non-contrast images. In conclusion, the DL model that integrates CTA imaging and clinical information can significantly improve the diagnostic accuracy and efficiency of AMI. Our approach has the potential to reduce the high mortality associated with AMI, ultimately leading to better clinical outcomes for patients facing this life-threatening condition.

## Data Availability

The raw data supporting the conclusions of this article will be made available by the authors, without undue reservation.
